# Effects of ketogenic diet on weight loss parameters among obese or overweight patients with polycystic ovary syndrome: a systematic review and meta-analysis of randomized controlled trails

**DOI:** 10.29219/fnr.v68.9835

**Published:** 2024-03-12

**Authors:** Nan-nan Xing, Fang Ren, Hui Yang

**Affiliations:** Department of Obstetrics and Gynecology, Shengjing Hospital of China Medical University, Shenyang, China

**Keywords:** ketogenic diet, weight loss, obesity, polycystic ovary syndrome, meta-analysis

## Abstract

**Aim:**

To evaluate how effective a low carbohydrate ketogenic diet (KD) is for changing key physical measurements such as weight, waist circumference (WC), body mass index (BMI), and fat mass (FM) in women with polycystic ovary syndrome (PCOS) who were obese or overweight.

**Methods:**

Several online databases, including PubMed, Scopus, EMBASE, Cochrane Library, and Web of Science (WOS), were searched systematically to find relevant randomized controlled trials (RCTs) up until June 2023. The Q-test and *I*^2^ statistics were used to assess the level of heterogeneity among the included studies. The data were then combined using either a fixed or random effects model and presented as a weighted mean difference (WMD) along with a 95% confidence interval (CI).

**Results:**

Of the 682 citations, 11 RCTs were included. The pooled results showed a significant decrease in the WMD of weight levels [WMD = −9.13 kg; 95% CI, −11.88, −6.39, *P* < 0.001; *I*^2^ = 87.23%] following KD. Moreover, KD significantly reduced BMI levels [WMD = −2.93 kg/m^2^; 95% CI, −3.65, −2.21, *P* < 0.001; *I*^2^ = 78.81%] compared to the controls. Patients with PCOS received KD demonstrated significant decrease in WC [WMD = −7.62 cm; 95% CI, −10.73, −4.50, *P* < 0.001; *I*^2^ = 89.17%] and FM [WMD = −5.32 kg; 95% CI, −7.29, −3.36, *P* < 0.001; *I*^2^ = 83.97%].

**Conclusion:**

KD was associated with lower weight loss (WL) parameters, including weight, BMI, WC, and FM, in obese or overweight women with PCOS, highlighting the significance of physicians and nurses in taking care of the nutritional needs of overweight/obese patients with PCOS.

## Popular scientific summary

- This meta-analysis of 11 RCTs examined how a low-carbohydrate ketogenic diet (KD) affects weight loss factors, such as weight, WC, BMI, and fat mass (FM), in women with PCOS who were obese or overweight.
- The pooled results indicated that the KD intervention led to significant reductions in body weight, BMI, WC, and FM.
- Further RCTs with larger sample sizes are necessary to authenticate the evidence and explore the most favorable dietary regimens.

Polycystic ovary syndrome (PCOS) is a common, complex hormonal and metabolic condition of unknown cause. It affects 5–20% of women of reproductive age worldwide depending on how it is diagnosed (1, 2). Women with PCOS typically exhibit oligoanovulation, biochemical or clinical hyperandrogenism, and/or polycystic ovarian morphology. Consequently, patients may present with symptoms such as infertility, insulin resistance (IR), hirsutism, acne, and dyslipidemia (3). Patients with PCOS are at risk for a range of medical complications throughout their lives, including obesity, metabolic dysfunction, vascular dysfunction, malignancy, reproductive complications, and mood disorders (4).

The treatment of PCOS typically involves a series of lifestyle modifications such as adjustments in diet, physical exercise, and weight management. Of these measures, weight loss (WL) is considered one of the most efficient strategies for regulating the menstrual cycle and alleviating PCOS symptoms (5, 6). The ketogenic diet (KD) is a dietary approach distinguished by its low-carbohydrate, high-fat, and moderate protein composition, which induces a state of nutritional ketosis through the elevated production of ketones, predominantly β-hydroxybutyrate and acetoacetate. Notably, KD has been found to have anti-seizure properties and is used to treat epilepsy that is difficult to manage (7). Research suggests that it may also be a useful treatment for other neurological conditions such as Parkinson’s disease, autism, and Alzheimer’s disease (8).

Moreover, previous clinical trials have reported beneficial therapeutic effects of KD in women diagnosed with PCOS by decreasing androgen secretion, elevating the circulating levels of sex hormone-binding globulin (SHBG), and improving insulin sensitivity (9). Furthermore, the endocrine and metabolic effects of KD are primarily characterized by a considerable decline in fasting serum insulin, percentage of free testosterone, weight, and luteinizing hormone (LH)/follicle-stimulating hormone (FSH) ratio in females with PCOS and obesity (10). An exceptional effect of KD in PCOS is the activation of AMPK and SIRT1, despite not being a caloric deprivation diet (11, 12). Upon activation, both AMPK and SIRT1 positively influence glucose homeostasis and enhance insulin sensitivity (12, 13).

Several meta-analyses have evaluated the impact of nutritional interventions on various aspects of PCOS, including biochemical factors (14–16), androgenic profiles (17, 18), and IR (19–22). However, no published meta-analysis has assessed the effect of KD on WL parameters in obese or overweight patients with PCOS. Therefore, this meta-analysis aimed to evaluate the efficacy of KD on WL parameters, including weight, waist circumference (WC), body mass index (BMI), and fat mass (FM), in obese or overweight women with PCOS.

## Method

### Literature search strategy

The PRISMA guidelines were followed in this systematic review and meta-analysis (23). We searched online databases such as PubMed, Web of Science (WOS), Scopus, Cochrane, and EMBASE to find relevant literature on the impact of the KD (carbohydrates less than 50 g/day) on WL parameters. We focused on clinical trials published until June 2023. For the initial data gathering, we used the following search strategy: (‘Diet, Ketogenic’ [MeSH] OR ‘Ketogenic*’[Title/Abstract]) AND (‘Polycystic Ovary Syndrome’[MeSH] OR ‘Polycystic Ovary Syndrome’[Title/Abstract] OR PCOS‘[Title/Abstract]) AND (‘Weight Loss’ [MeSH] OR ‘Weight Loss*’[Title/Abstract] OR ‘Body Mass Index’[MeSH] OR ‘Body Mass Index’[Title/Abstract] OR ‘BMI’[Title/Abstract] OR ‘Obesity’[MeSH] OR ‘Obesity’[Title/Abstract] OR ‘Overweight’[MeSH] OR ‘Overweight’[Title/Abstract] OR ‘waist circumference’[MeSH] OR ‘waist circumference’[Title/Abstract] OR ‘Body Mass*’[Title/Abstract] OR ‘Fat Mass’[Title/Abstract]). A manual search of the references in the selected articles and previous reviews was conducted to identify additional relevant articles. The titles and abstracts of the included trials, data extraction, and evaluation of eligible studies were conducted separately by two authors (FR and HY). Any discrepancies were resolved by consultation with a third author (NX) to ensure accuracy.

### Criteria for inclusion and exclusion

This review included clinical trials that met the following criteria: 1) trials with crossover, parallel, or before-after designs involving overweight or obese women with PCOS; 2) trials that investigated the effects of KDs (carbohydrates less than 50 g/day) on WL parameters, such as WL, BMI, WC, and FM; and 3) articles published in English. Trials that did not report mean differences in WL parameters along with standard deviation (SD), *in vitro* studies, abstracts of seminars without full text, or case reports or letters to editors were excluded from the current meta-analysis.

### Extraction of data and quality assessment

Two reviewers individually conduct the quality assessments and data extraction for the included trials. The National Institutes of Health (NIH) and the Cochrane Collaboration Risk of Bias tools were used for quality assessment of the included trials with RCT and before-after design, while a standard Excel sheet was used for data extraction. The methodological quality of the clinical trials included in this review was assessed based on criteria such as ‘blinding of participants and outcome assessors, randomization generation, allocation concealment, selective outcome reporting, incomplete outcome data, and other sources of bias’. The following data were extracted: first author’s name, age of participants, study location, publication year, study method, total sample size, type and duration of intervention, and mean (SD) changes in WL factors (WL, BMI, WC, and FM) for women with PCOS with and without KDs.

### Statistical analysis

STATA V.11.0 and Comprehensive Meta-Analysis V2 (CMA V2) were used for all statistical analyses. The effects of KDs on changes in WL factors were determined, and pooled effect sizes were calculated using weighted mean differences (WMDs) with a 95% confidence interval (CI). The following formulae were used for the calculations: [meanpost – meanpre] for the mean change and [√([SDpost2 + SDpre2] – [2 × R × SDpost × SDpre])] for the corresponding SD. A correlation coefficient with a value of 0.8 was postulated for the variable ‘R’ in the computation. The evaluation of heterogeneity within the studies incorporated was conducted through the application of statistical tests, namely, the Cochran (Q) and I-squared (*I*^2^) tests. If the value of *I*^2^ surpassed 50% and a P-value less than 0.1 was obtained, then the application of the random-effects model was applied. Alternatively, if the aforementioned criteria were not met, then the fixed-effects model was employed. Subgroup analyses were done to detect possible sources of heterogeneity based on the moderator variables. Sensitivity analyses were performed to evaluate the impact of each trial on the dependability of the combined WMDs by means of the leave-one-out method. Quantitative and visual assessments were conducted to evaluate potential evidence of publication bias using funnel plot and Egger’s test. The level of statistical significance was established as *P* < 0.05.

## Results

**Fig. 1:**
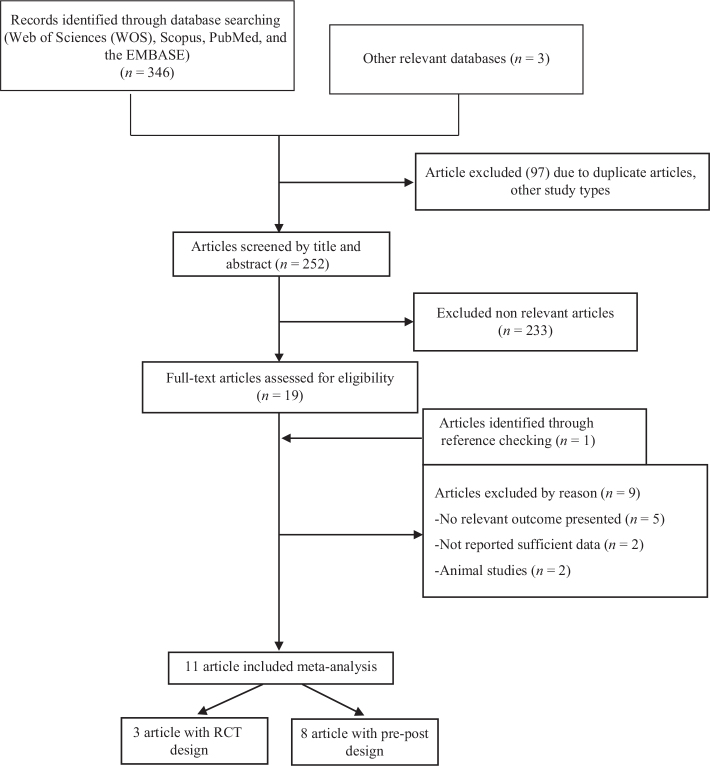
Flowchart study identify and study selection.

After eliminating duplicate and irrelevant studies, 11 articles (comprising 12 trials) were selected for the present meta-analysis out of the initial 349 citations. The systematic procedure of identifying relevant studies is illustrated in Fig. 1.

Among the included articles, three were randomized, double-blind, placebo-controlled trials, whereas the remaining eight were before-after studies. This meta-analysis included 426 women with PCOS. Yang et al. (24) conducted a study that examined different disease types, specifically PCOS with non-hyperuricemia and hyperuricemia, and treated each disease type as a separate study within the meta-analysis. The intervention duration across the included studies varied from 6 to 24 weeks. The selected trials were published between 2005 and 2023. Table 1 elucidates the characteristics of the included trials.

**Table 1:** Main characteristic of included studies

Authors/country	Study design	Total sample size	Interventions	Duration (weeks)	Outcomes
Li et al. (25)/China	RCT	18	The KD was composed of approximately 5–10% energy derived from carbohydrates, which equates to a maximum of 50 g/day. Protein intake accounted for 18–27% of energy, while the majority of energy, approximately 70–75%, was obtained from fat.	12	WL, BMI (Patients: 29.82 ± 2.36; Control: 32.81 ± 3.75), and FM
Avolio et al. (26)/Italy	Before-after trial	57	A ketogenic diet was defined as a carbohydrate intake below 20 g/day. The ketogenic diet aimed to achieve a daily energy intake of 750–800 calories, with 35–40% of the calories coming from proteins (equivalent to 1.2 g per kilogram of ideal body weight). Fat intake accounted for 45–50% of calories, with less than 10% of calories derived from saturated fat. Carbohydrate intake was limited to 10% of the total calories, which equated to less than 20 g/day.	24	FM
Cincione et al. (27)/Italy	Before-after trial	17	The maximum daily carbohydrate intake was limited to 30 g/day. Additionally, the total daily caloric intake was set at approximately 600 calories. The diet employed in the study was classified as a very low-calorie ketogenic diet (VLCKD).	6	WL, BMI (31.84 ± 5.85 kg/m^2^), WC, and FM
Cincione et al. (28)/Italy	RCT	144	The daily intake of carbohydrates was restricted to a maximum of 30 g/day, and the total daily caloric intake was set at approximately 600 calories. The diet implemented in this study was categorized as a very low-calorie ketogenic diet (VLCKD).	6	WL, BMI (KD: 34.69 ± 5.26; Mediterranean: 33.96 ± 4.72), WC, and FM
Mavropoulos et al. (29)/USA	Before-after trial	5	Throughout the 6-month study period, a low-carbohydrate ketogenic diet (LCKD) was implemented, with an intake of less than 20 g of carbohydrates per day, as tolerated by the participants.	24	WL
liran et al. (30)/China	Before-after trial	10	The ketogenic diet (KD) followed a daily carbohydrate intake of less than 50 g.	4	WL and BMI (27.1 ± 3.0)
Jian et al. (31)/China	Before-after trial	51	The KD included a daily carbohydrate intake of 50 g or less.	4	WL and BMI (29.87 ± 3.79)
Pandurevic et al. (32)/Italy	RCT	30	During the very-low-calorie ketogenic diet (VLCKD) phase, there were three steps, with daily caloric intake ranging from 600 to 800 kcal. The diet primarily included high-biological-value protein preparations derived from sources such as cow milk, soy, eggs, green peas, and cereals. Carbohydrate intake obtained from vegetables was restricted to less than 50 g/day. Additionally, the diet included a daily intake of 10 g olive oil.	16	BMI (Experimental: 33.9 ± 3.8; Control 33.7 ± 4.3), WC, and FM
Magagnini et al. (33)/Italy	Before-after trial	25	In the VLCKD protocol, the diet includes high-biological proteins obtained from sources, such as milk, peas, and whey. Each protein preparation, with an approximate caloric content of 100–150 kcal, contained 18 g of protein, 4 g of carbohydrates, and 3 or 4 g of fat. This composition was consistent with the VLCKD requirements.	12	BMI (32.8 ± 1.0) and WC
Paoli et al. (12)/Italy	Before-after trial	14	A modified KD protocol was employed, which involved the use of formulas with high protein content (19 g per portion) and very low carbohydrate content (3.5 g per portion). This modified protocol aimed to maintain the desired macronutrient ratios for effective ketogenic diet implementation.	12	WL, BMI (28.84 ± 2.10), WC, and FM
Yang (a) et al. (24)/China	Before-after trial	27	The ketogenic diet had a recommended carbohydrate intake of less than 50 g/day. The energy supply ratio for the three macronutrients in the ketogenic diet was approximately 70–75% fat, 5–10% carbohydrate, and 18–27% protein.	12	WL
Yang (b) et al. (24)/China	Before-after trial	28	The ketogenic diet had a recommended carbohydrate intake of less than 50 g/day. The energy supply ratio for the three macronutrients in the ketogenic diet was approximately 70–75% fat, 5–10% carbohydrate, and 18–27% protein.	12	WL

### Main outcomes

**Fig. 2:**
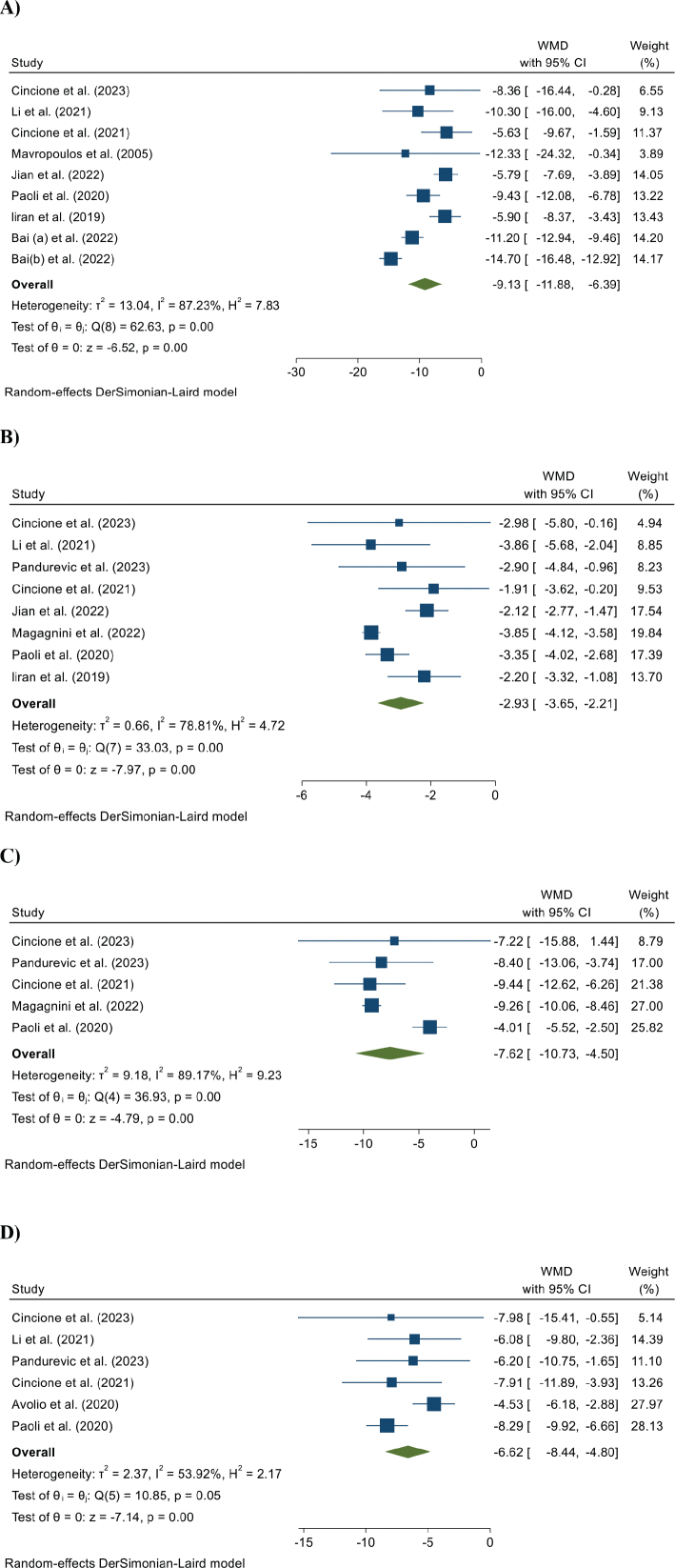
The effects of a ketogenic diet on (A) weight, (B) BMI, (C) WC, and (D) FM levels in overweight or obese women with PCOS.

Figure 2 shows forest plots illustrating the effects of KDs on the WL parameters. The pooled meta-analysis, utilizing a random-effect model, revealed significant reductions in weight (WMD = −9.13 kg; 95% CI, −11.88 to −6.39; *P* < 0.01; *I*^2^: 87.23%), BMI (WMD = −2.93 kg/m^2^; 95% CI, −3.65 to −2.21; *P* < 0.01; *I*^2^: 78.81%), WC (WMD = −7.62 cm; 95% CI, −10.73 to −4.50; *P* < 0.01; *I*^2^: 89.17%), and FM (WMD = −6.62 kg; 95% CI, −8.44 to −4.80; *P* < 0.01; *I*^2^: 53.92%).

### Sensitivity analyses and subgroup analysis

Following the sensitivity analysis, our findings exhibited that there were no significant deviations between the pre- and post-sensitivity WMDs for variables such as weight, WC, BMI, and FM. However, when excluding the study by Jian et al. (31), the sensitivity analysis showed a lower pooled WMD of −9.69 (95% CI: −12.45, −6.93) for weight, and when excluding the study by Yang (b) et al. (24), the higher pooled WMD was −8.12 (95% CI: −10.28, −5.96). For BMI, the lower pooled WMD in sensitivity analysis was −3.17 (95% CI: −3.79, −2.55) after excluding Jian et al. (31), and the higher was −2.68 (95% CI: −3.27, −2.09) after removing Magagnini et al. (33). Regarding WC and FM, the lower pooled WMD in sensitivity analysis was −9.23 (95% CI: −9.99, −8.46) and −7.78 (95% CI: −9.10, −6.47), respectively, after excluding studies by Paoli et al. (12) and Avolio et al. (26). The higher pooled WMDs in sensitivity analysis were −6.98 (95% CI: −10.51, −3.46) and −6.42 (95% CI: −8.51, −4.34) after removing studies by Magagnini et al. (33) and Cincione et al. (27), respectively.

**Fig. 3:**
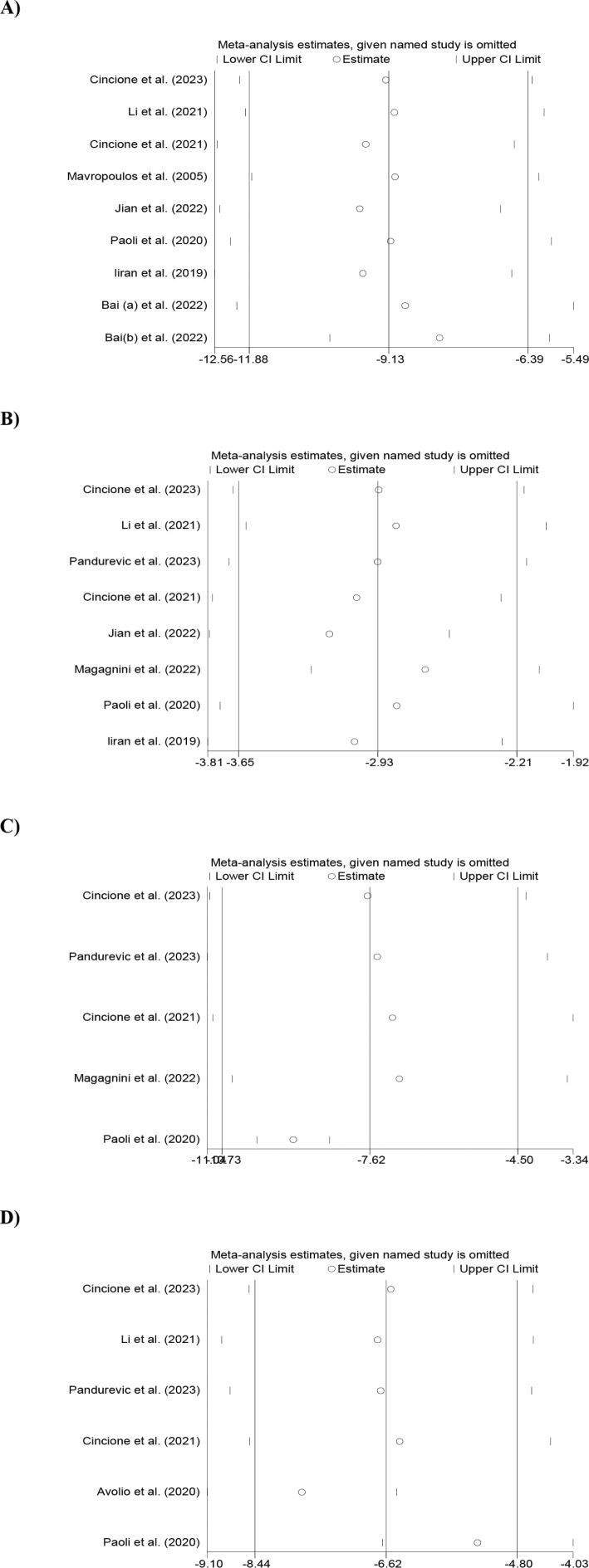
Sensitivity analyses findings of the effects of a ketogenic diet on (A) weight, (B) BMI, (C) WC, and (D) FM levels in overweight or obese women with PCOS.

Figure 3A–D depicts the outcomes of the sensitivity analysis conducted subsequent to the exclusion of each trial from the meta-analysis. When we conducted a subgroup analysis according to the duration of the intervention (≥12 weeks vs. <12 weeks), noteworthy reductions in WL factors were observed in studies with intervention durations of ≥12 weeks and <12 weeks.

### Publication bias and quality assessment

Upon performing quantitative and visual assessments, we did not find any significant evidence of publication bias. However, the P-values obtained from Egger’s test were 0.23 for weight, 0.96 for BMI, 0.91 for WC, and 0.76 for FM (Supplementary Fig. S1–S4). All included trials with RCT design were of low quality using the Cochrane Collaboration Risk of Bias tool, and 3 of 5 before and after articles were of high quality using the NIH tool (Supplementary Tables S1–S2).

## Discussion

To the best of the authors’ knowledge, this is the first systematic review and meta-analysis that investigated the effect of KDs on body composition in obese or overweight PCOS patients over 11 included RCTs. Pooled findings revealed that body weight, BMI, WC, and FM significantly decreased following KD intervention.

Research is growing on how adjusting diets can help treat PCOS. When carbohydrate intake is reduced and fat supply is improved, the body will start metabolizing fat for energy. KDs mimic the metabolic state of fasting and have been proven in several RCTs to decrease obesity in participants. Body weight, BMI, FM, and WC could be significantly reduced (34–36). Patients diagnosed with non-alcoholic fatty liver disease (NAFLD) who followed a KD also lost significant amounts of weight, with reductions in visceral adipose tissue and liver fat fraction compared to those on a standard diet (37). A recent meta-analysis of 10 RCTs assessed the effect of KD on body composition and metabolic parameters in patients with cancer. Their pooled effect sizes demonstrated a significant decrease in body weight and FM post KDs intervention (38). KDs could potentially aid in reducing body fat and visceral adipose tissue without affecting lean body mass (39, 40). Moreover, a recently published meta-analysis of 21 clinical trials involving obese/overweight patients with T2DM found that the implementation of a low-carbohydrate KD resulted in significant decreases in body weight, BMI, and WC. Additionally, non-diabetic patients experienced significant decreases in body FM. The findings of this study suggest that such KDs could be beneficial for obese and overweight patients with T2DM, as it can improve cardiovascular risk factors such as weight, glycemic, and lipid profile (41). Our meta-analysis has established that KDs have the potential to reduce the weight, WC, BMI, and FM of patients with PCOS.

PCOS often leads to weight gain and central obesity, which typically appear before the onset of anovulatory menstrual cycles (42). In these cases, visceral adiposity is linked to increased IR, which worsens both metabolic and reproductive disorders. Obesity also has a significant effect on the PCOS phenotype as it is linked to a greater occurrence of menstrual irregularities, hyperandrogenemia, and hirsutism (26, 43). The etiology of PCOS remains uncertain; however, IR appears to be a significant contributing factor. Women diagnosed with PCOS frequently exhibit concomitant IR, which can be attributed to excessive weight and obesity (26). Recent reproductive and endocrine studies have highlighted a significant role of diet in IR. Studies have suggested that specific well-balanced diets, including low glycemic index (GI) diets, Mediterranean diets, Dietary Approaches to Stop Hypertension (DASH) diets, and vegetarian diets, can improve IR, regulate metabolism, manage body weight, and prevent related complications (44–46). Despite these recommendations, PCOS patients are often hesitant to follow the guidelines, indicating the importance of dietary and exercise interventions as first-line management for these patients (47), or adopting self-help methods (48, 49).

Adipose tissue serves as an additional source of androgens, and its excessive accumulation can exacerbate hyperandrogenism (50). Adipocytes located in the abdominal region exhibit greater activity as endocrine cells than those found in the lower body, which characterizes gynoid obesity (51). These cells have a greater sensitivity to catecholamines and a reduced sensitivity to insulin, leading to an increase in insulin production and mild inflammation, changes in lipid levels, higher levels of androgen production, and lower levels of SHBG. These factors collectively promote anovulation (12). Women with PCOS who exhibit hyperandrogenism, high BMI, and IR have diminished metabolic flexibility (as determined by changes in respiratory quotient following insulin stimulation) (52, 53). The present meta-analysis also demonstrated a significant reduction in WL and BMI in PCOS patients.

Guidelines for treating PCOS recommend lifestyle changes as an initial therapeutic approach. This involves modifying diet and increasing physical activity, which have been found to be effective in achieving WL and altering body composition (27). The present meta-analysis confirms previous reports that a low-calorie diet with a high nutritional value is effective for managing PCOS. Most of the included studies reported a carbohydrate intake lower than 20 g in the KD, which improves insulin sensitivity by reducing carbohydrates and calories. KD therapy also personalized protein intake to maintain lean mass for sustained results (54, 55). The lipid supply is rich in mono- and polyunsaturated fatty acids that are known for their insulin-sensitizing effects, as well as their anti-inflammatory actions (56). However, the long-term effects of this therapy need to be confirmed through follow-up studies to determine whether sustained results can be achieved with less restrictive, more sustainable eating behaviors.

Our meta-analysis differs from previous studies because it included more newly published studies and statistics. This meta-analysis had certain limitations. First, blinding of the outcome assessment was not possible because dietary modification was the only intervention. Moreover, dietary regulation was not strictly implemented, and ingredients varied, which may have adversely affected the accuracy of the results. Second, heterogeneity existed in our analysis owing to differences in patient physical characteristics. Third, a potential limitation of this study is that some of the studies evaluated had a before-and-after design without a control group, which may affect the reliability of the overall results. Finally, although we gathered a substantial number of RCTs, these are still insufficient to reach a definitive conclusion on the benefits of KDs. It is essential to conduct more RCTs to support our findings, especially since most studies have focused on interventions lasting less than 6 months. Future research should examine whether these effects can be maintained over a longer period. Moreover, given the proficiency and responsibility of nurses in catering to the nutritional demands of patients, it becomes imperative for them to engage in nutrition screening and offer suitable dietary guidance, such as the KD, to enhance the adoption of wholesome eating habits and consequently ameliorate the body weight outcomes of obese or overweight patients with PCOS.

## Conclusion

Dietary modifications may contribute to the amelioration of weight management in females afflicted with PCOS. KD might be considered an ideal option for enhancing weight management, as it was shown by decreasing body weight, BMI, WC, and FM in obese or overweight PCOS patients. Overall, dietary modifications constitute an efficacious, tolerable, and secure methodology, thereby providing viable alternatives for patients who are unable to cope with the gastrointestinal side effects of metformin. Nevertheless, given the limited number of studies and the small sample sizes observed in certain included studies, it is imperative to interpret the findings cautiously. Additional RCTs featuring robust designs and large sample sizes are warranted to validate the evidence and investigate optimal dietary patterns.

## Data Availability

All data generated or analyzed during this study are included in this article.
